# Evidence of a Viable but Nonculturable (VBNC) Phase in *B. abortus* S19 under Oxidative Stress (H_2_O_2_, -Fe^2+^, Bleach) and under Non-Oxidative Inhibitory Conditions (Isopropanol, Erythritol, Selenite)

**DOI:** 10.3390/microorganisms12030491

**Published:** 2024-02-28

**Authors:** Jens Jacob

**Affiliations:** Unit 14 Hospital Hygiene and Infection Prevention and Control, Robert Koch Institute, Nordufer 20, 13353 Berlin, Germany; jacobj@rki.de; Tel.: +49-18754-2667

**Keywords:** *Brucella*, VBNC, oxidative stress, charcoal agar

## Abstract

The effect of oxidative stress on the survival of various *Brucella* species has not been fully investigated yet. We here conducted a study in which we investigated the effect of different types of oxidative stress (Fe^2+^, H_2_O_2_, bleach) versus non-oxidative inhibitory effects (selenite, erythritol, and isopropanol) on the survival of *B. abortus* S19, *B. abortus* S19 ∆mglA 3.14, and *B. neotomae* 5K33. The work focuses on the appearance of ATP–CFU quotient imbalances indicating the existence of a viable but nonculturable (VBNC) form of *B. abortus* S19, as has previously been shown.

## 1. Introduction

Bacteria of the genus *Brucella* are α-proteobacteria. They are phylogenetically closely related to plant pathogens and symbionts (e.g., *Rhizobium* and *Agrobacterium*) as well as to intracellular animal parasites like *Bartonella* and to opportunistic bacteria of the genus *Ochrobactrum*. Oxidative stress, caused by Fe^2+^, H_2_O_2_, or bleach acts via release of active oxygen radicals (O_2_^−^, OH^−^). Investigations into the effects of Fe^2+^ in the presence (Fenton effect) or absence of H_2_O_2_ on the survival of various *Brucella* species have already started [1,2].

As for non-oxidative stress, erythritol inhibits *B. abortus* S19 as a result of defective catabolism i.e., accumulation of toxic erythritol metabolism products. Previously, the products of the ERY operon were identified as an erythritol kinase (*eryA*), two putative dehydrogenases (*eryB*, *eryC*), and a repressor gene (*eryD*). *B. abortus* S19, which has been extensively used for vaccination of cattle against brucellosis, was spontaneously attenuated by a mutation deleting the 3′ end of *eryC* and the 5′ region of *eryD* genes, leading to a fused *eryC-D* polypeptide. Stress in *B. abortus* S19 is caused by accumulation of 3-keto-l-erythrose 4-phosphate [3,4].

The non-oxidative stress noxe selenium is widely known as an essential element acting as the central atom in glutathionperoxidase. However, excess of selenium causes competitive exclusion of S (sulfur) from incorporation into essential amino acids methionine and cysteine, leading to the mis-formation of selenomethionine and selenocysteine.

The non-oxidative isopropanol in aqueous systems causes the de-arrangement of lipids and, respectively, the precipitation of nucleic acids.

Viable but nonculturable (VBNC) form bacteria have previously been described for *Camplyobacter* [5,6], *Helicobacter* [7,8], *Franchisella* [9], *Pseudomonas* [10], and also for close relatives of *Brucella*, *Rhizobiae*, and *Agrobacterium* [11,12].

Because the first information about a VBNC *Brucella* resulted from our previous study [1], we here conducted a study in which we investigated the effects of iron, H_2_O_2_, and bleach, respectively, on the survival of *B. abortus* S19, *B. abortus* S19 ∆mglA 3.14, and *B. neotomae* 5K33, including in comparison to non-oxidative inhibitory substances, with special attention given to ATP–CFU imbalances.

In the study presented here, a significantly higher ATP–CFU ratio was described, concluding to the existence of a viable but nonculturable (VBNC) form of *B. abortus* S19.

The presented data point to a VBNC state of *B. abortus* S19 after exposure to oxidative stress. In order to investigate this phenomenon in detail, we conducted experiments using various forms of oxidative stress and different media for re-cultivation of *B. abortus* S19, *B. abortus* ∆mglA, and *B. neotomae*.

As the main tool for obtaining evidence for this VBNC form, we used different plating on a TSA and on a newly developed, charcoal-based (stress-scavenging) agar medium (CYE). However, the main indicator for the existence of this VBNC is a quotient that puts the ATP-RLU and the CFU counts in relation. This is based on the general assumption by the developers of the BAC Titer Glow^®^ Kit (PROMEGA, Madison, WI, USA) that one bacterium possesses (by their definition) one energy unit/one organism; i.e., the measured ATP-RLU LUMI counts divided by the CFU/mL counts should be as per definition 1: “one standard energy unit per bacterium”.

If these quotients extend beyond 1, this is an indicator that not all bacteria are cultivable at that specific moment (partly in VBNC form) because one bacterium can possess only one energy unit. The parallel plating of both the CYE (charcoal based) agar indeed recovered such bacteria as were cultivable and which were not found growing on the TSA.

On the other hand, if these quotients drop toward 0, that indicates that the organism is running out of energy. For example, a quotient of 0.02 would mean that only 2% of the (“1”) standard energy unit, i.e., 100% standard energy content/bacterium, was detected.

## 2. Material and Methods

### 2.1. Bacterial Strains

*B. abortus* S19 [13], *B. abortus* ∆mglA 3.14 [14], *B. neotomae* 5K33 [15], *B. microti* CCM4915 [16], and *Brucella* 83/13 [17] were grown in tryptic soy broth (TSB) and on the activated-charcoal-based *Brucella* agar medium, CYE agar. Ingredients per liter were 35 g BCYE (buffered charcoal yeast extract agar, Fluka, art.-nr. 78123-55G), 17 g casein peptone, 5 g NaCl, and 5 g soybean peptone.

### 2.2. Survival Experiments under Oxidative Stress

For comparison of the effects of certain oxidative stress noxes in contrast to non-oxidative stress, the “not-oxidative” inhibiting substances erythritol and isopropanol were used. The respective substances were 0.2 mg/mL (1.6 mM) Fe^2+^, 0.5% H_2_O_2_, 0.5% bleach, 10 mM selenite, 10 mM erythritol, 70% isopropanol.

The experiments for testing oxidative stress were carried out in standardized aqueous systems [1].

For performing the assays, bacterial cells from −80 °C stocks with standardized viable cell counts of *B. abortus* S19, *B. abortus* S19 ∆mglA, *B. neotomae* 5K33, *Brucella* ssp. 83/13, and *B. microti* CCM4915, respectively, were used to yield initial experimental inoculums of 1 × 10^6^ cells/mL. Survival of cells was determined by taking aliquots of 100 μL each and plating on tryptic soy agar (TSA) and the new *Brucella* medium CYE, respectively.

### 2.3. Determination of Viability of Bacteria

#### 2.3.1. Determination of Colony Forming Units (CFU)

For counting the colony forming units (CFU), defined volumes of bacteria were plated on tryptic soy agar (TSA) and the *Brucella* (CYE) agar, respectively. Serial dilutions were prepared, plated, and incubated for up to 3 days at 37 °C.

#### 2.3.2. Determination of Intracellular ATP Content

ATP luminescence was measured with a BacTiter-Glo^®^ microbial viability assay (PROMEGA, cat. nr. G8231, Madison, WI, USA). All experiments were carried out in white, 96-well microplate dishes designed for luminescence measurements (THERMOSCIENTIFIC, cat. nr. 236105, Waltham, MA, USA).

The BacTiter-Glo^®^ reagent was used according to the manufacturer’s manual.

For the setup of the experiments, the substances to be examined were 0.2 mg/mL (1.6 mM) Fe^2+^, 0.5% H_2_O_2_, 0.5% bleach, 10 mM selenite, 10 mM erythritol, 70% isopropanol.

They were spotted on the first row of the microtiter plate in the respective standardized aqueous systems and then diluted to 100 µL volume per well. Subsequently, the wells were inoculated with 10 µL of a suspension of bacteria, so that a concentration of 1 × 10^7^ bacteria/mL was achieved. Experiments were performed as triplicates and a blank without inoculation of bacteria was carried out in parallel.

After 30 min. of aerobic incubation, 100 µL of freshly prepared Bac Titer-Glo^®^ reagent was added and the plate covered with Adhesive Film^®^ (NEOLAB, cat. nr.7-5170). In this step, the bacteria were lysed and ATP was measured via the luminescence reaction using the TECAN Infinite 200 M Pro (TECAN, Gröding, Austria) reader and expressed as relative light units (RLU), as described in the manual of the BacTiterGlow*Kit (BacTiter-Glo^®^ microbial viability assay (PROMEGA, cat. nr. G8231, Madison, WI, USA), as outlined in detail previously (Jacob et al., 2020). For that, the “standard automatic” luminescence measurement program was used.

The ascertained RLU values of the bacteria were considered to be 100%. Colony forming units (CFUs) were determined in parallel by plating on TSA and CYE. They were counted after 3 days. In the case of the H_2_O_2_ experiments, the samples were subjected to neutralization with catalase (25 µg/mL). All luminescence experiments were repeated at least three times.

#### 2.3.3. Membrane Integrity

The Live/Dead BacLight^®^ bacterial viability kit (LIVE/DEAD BacLight^®^ bacterial viability kit, cat. nr. L7012, MOLECULAR PROBES, Eugene, OR, USA) was used to test bacterial membrane integrity on the basis of the interaction of two fluorescing dyes, Syto 9 and propidium iodide (PI).

Syto 9 is a green fluorescing dye and stains both viable and dead cells. Propidium iodide (PI) is a red fluorescing dye and stains nucleoli and chromosomal structures. PI is used for staining dead cells within a population of cells because it can penetrate only into cells with damaged membranes.

### 2.4. Statistics

Graph Pad Prism 7.04 (San Diego, CA, USA) was used for statistical analysis (Mann–Whitney U test) of the data. Data of the survival assays were based on at least three independent experiments each using triplicates.

## 3. Results

### 3.1. Survival of Brucella in Fe^2+^ as Determined by CFU and ATP Content

In order to investigate the effects of Fe^2+^ on the survival of *B. abortus* S19, *B. abortus* S19 ∆mglA 3.14, and *B. neotomae* 5K33, we exposed these bacteria to Fe^2+^, as previously described [1].

**Figure 1:**
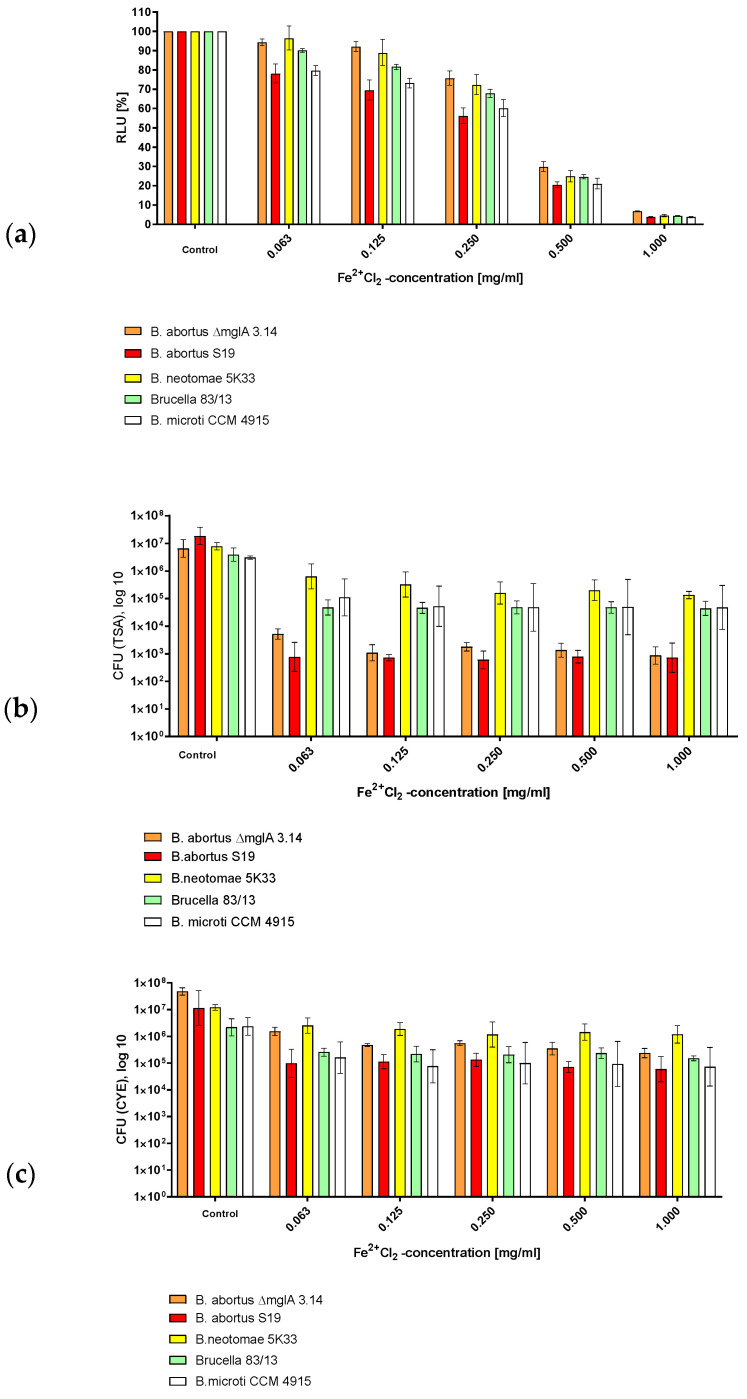
Susceptibility of *B. abortus* S19A, *B. abortus* ∆mglA 3.14, *B. neotomae* 5K33, *B. microti* CCM 4915, and *B.* 83/13 to Fe^2+^ after 30 min of exposition as determined via (**a**) ATP content, (**b**) CFUs on TSA, and (**c**) CFUs on CYE agar.

Exposure of the various *Brucellae* used in this investigation demonstrated that *B. neotomae* (1d) survived longer than *B. abortus* in this environment (Figure 1, Table 1). *B. abortus* S19 was most susceptible with respect to drop of luminescence, whereas both *B. abortus* S19 and *B. abortus* S19 ΔmglA showed a comparable loss of cultivability with respect to the TSA agar. A part of this cultivability loss was recovered via growth on CYE agar. Table 1 reports the luminescence and CFU values, in case of Fe^2+^ exposition, and their relation, i.e., quotas. The increase in the quotas indicates that relatively more ATP was measured in comparison to the CFU counts in the cases of *B. abortus* S19 and *B. abortus* S19 ΔmglA. Concluding from that finding, we postulate that some of the (still alive, energy-possessing) bacteria were driven to the VBNC phase. That was not shown for *B. neotomae* 5K33.

### 3.2. Survival of Brucella in H_2_O_2_ as Determined by CFU and ATP Content

In order to investigate the effect of H_2_O_2_ on the survival of *B. abortus* S19, *B. abortus* S19 ∆mglA 3.14, and *B. neotomae* 5K33, we exposed these bacteria to H_2_O_2_ as previously described [1].

**Figure 2:**
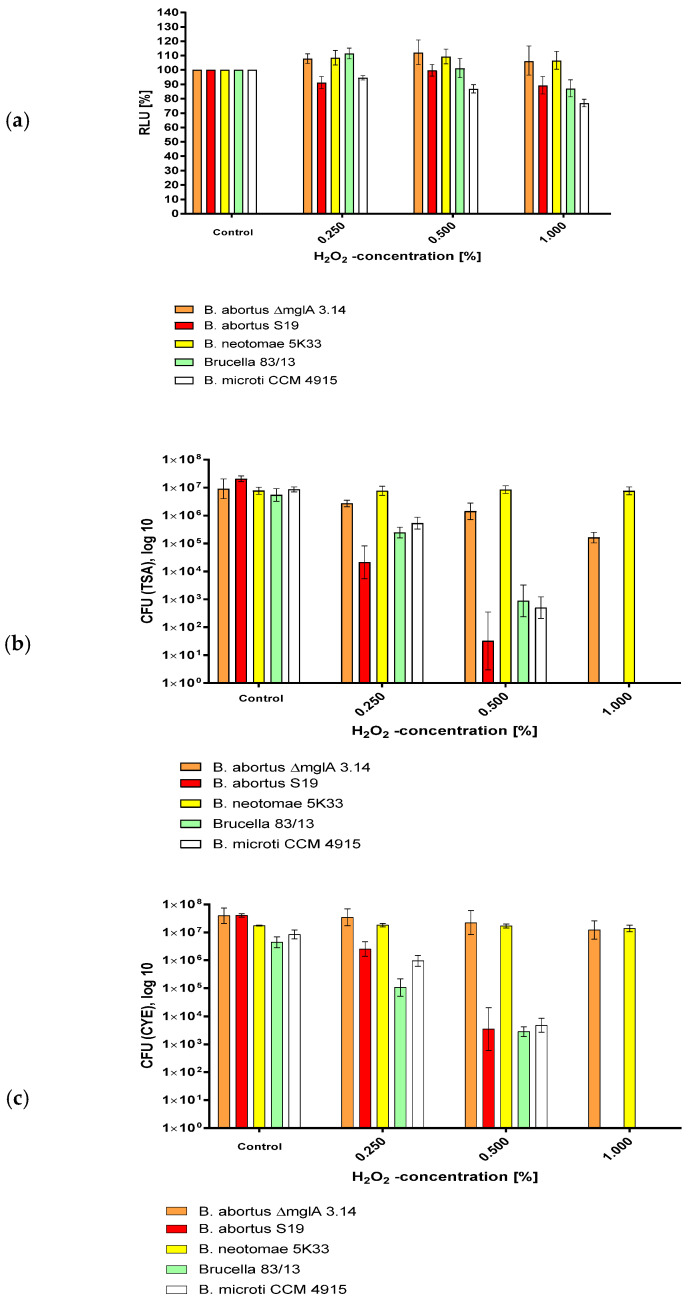
Susceptibility of *B. abortus* S19A, *B. abortus* ∆mglA 3.14, *B. neotomae* 5K33, *B. microti* CCM 4915, and *B.* 83/13 to H_2_O_2_ after 30 min of exposure as determined by (**a**) ATP content, (**b**) CFU (TSA), and (**c**) CFU (CYE).

The exposure of the various *Brucellae* used in this investigation demonstrated that *B. neotomae* (1d) survived longer than *B. abortus* in this environment (Figure 2, Table 2). *B. abortus* S19 was most susceptible with respect to drop of luminescence, whereas both *B. abortus* S19 and *B. abortus* S19 ΔmglA showed a comparable loss of cultivability with respect to TSA agar. Part of this cultivability loss was recovered by growth on CYE agar. Table 2 reports the luminescence and CFU values in the case of H_2_O_2_ exposure in terms of their relation, i.e., quotas. The quotas that increased that relatively more ATP was measured in comparison to the CFU counts in the cases of *B. abortus* S19 and *B. abortus* S19 ΔmglA. Concluding from that finding, we postulate that some of the (still alive, energy-possessing) bacteria were driven to the VBNC phase, which was not shown for *B. neotomae* 5K33.

The latter was not shown for 1% H_2_O_2_, indicating that in this case, the bacteria were really dead under the stress of this H_2_O_2_ concentration.

### 3.3. Survival of Brucella in a Laboratory Standard Environment Containing Bleach as Determined by CFU and ATP Content

**Figure 3:**
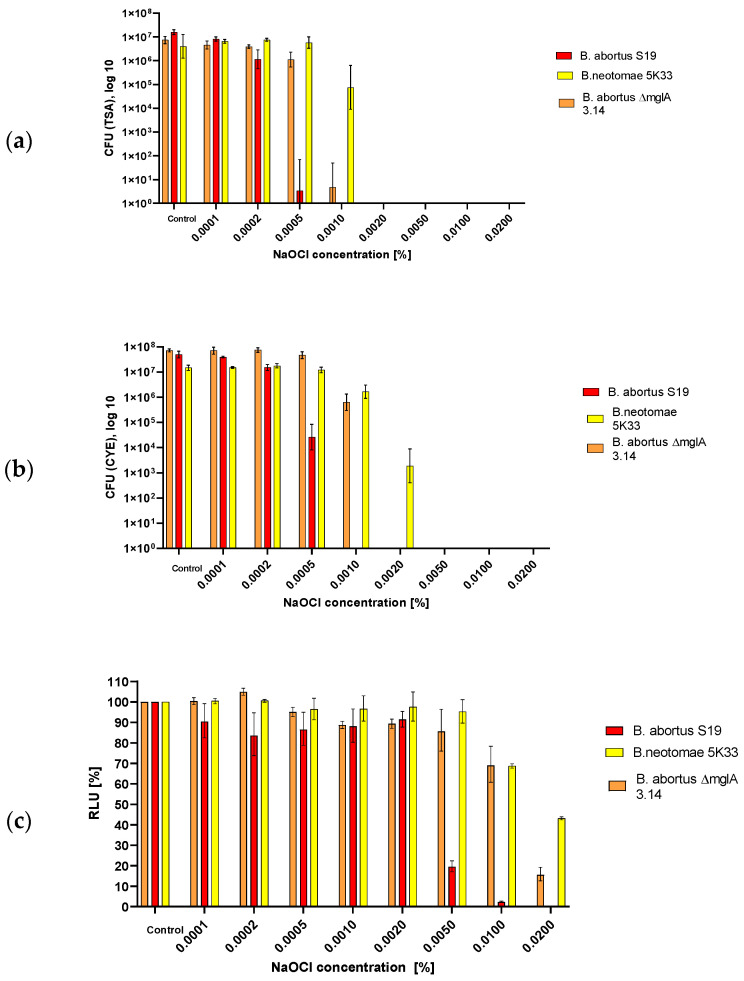
Effects of bleach on *B. abortus* S19A, *B. abortus* ∆mglA 3.14, and *B.neotomae* 5K33 after 30 min of exposure, as determined by ATP content (**c**), CFU on TSA (**a**), and CFU on CYE agar (**b**).

This standard laboratory environment included the use of bleach at pH 6. The exposition of the various *Brucellae* used in this investigation demonstrated that *B. neotomae* (1d) survived longer than *B. abortus* in this environment (Figure 3, Table 3). *B. abortus* S19 was most susceptible with respect to drop of luminescence, whereas both *B. abortus* S19 and *B. abortus* S19 ΔmglA showed a comparable loss of cultivability with respect to TSA agar. Part of the *B. abortus* S19 ΔmglA cultivability loss was recovered by growth on CYE agar. Table 3 reports the luminescence and CFU values in the case of bleach exposure according to their relation, i.e., quotas. The increased quotas indicate that relatively more ATP was measured in comparison to the CFU counts in the cases of *B. abortus* S19 and *B. abortus* S19 ΔmglA. Additionally, it is to be noted here that the effect of the bleach concentration used was so strong that no cultivability whatsover could be detected in some variants of the assay. However, concluding from these findings, we postulate too that some of the (still alive, energy-possessing) bacteria were driven to the VBNC phase, which was not shown for *B. neotomae* 5K33.

### 3.4. Effects of Selenite on CFU and ATP Content, Respectively, and ATP/CFU Quotients

**Figure 4:**
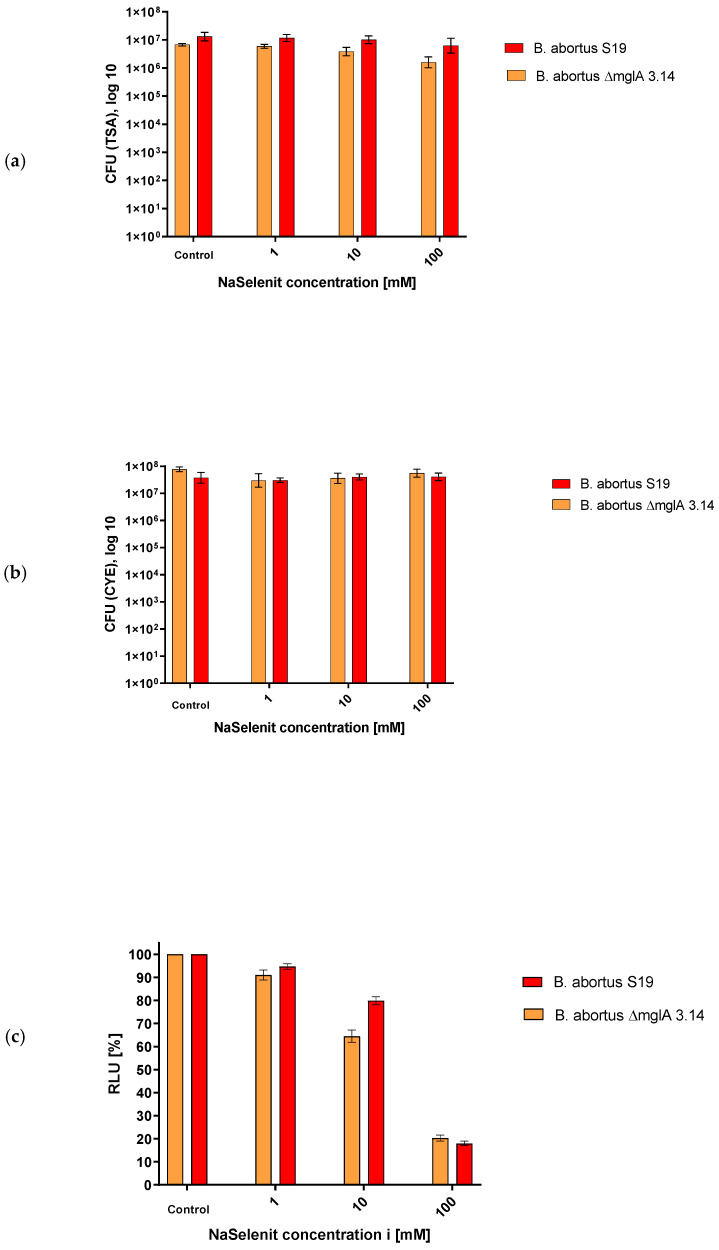
Selenite effect on *B. abortus* S19A and *B. abortus* ∆mglA 3.14 after 30 min of room temperature exposure as determined via ATP ontent (**c**), CFU on TSA (**a**), and CFU on CYE agar (**b**).

The non-oxidative effect of selenite on ATP content and cultivability on TSA and CYE is reported in Table 4 and Figure 4, showing a similar decline (but presence) across the strains tested. There was no visible difference in recovery with CYE in contrast to TSA agar. Table 4 reports the luminescence and CFU values in relation in the case of selenite exposure, i.e., quotas. No increase in quotas was seen. This indicates that relatively no more ATP was measured in comparison to the CFU counts in the cases of *B. abortus* S19 and *B. abortus* S19 ΔmglA. Concluding from that finding, we assume that under these conditions, the bacteria were not driven to the VBNC phase.

### 3.5. Effect of Erythritol on CFU and ATP Content and Effect on ATP/CFU Ratios

**Figure 5:**
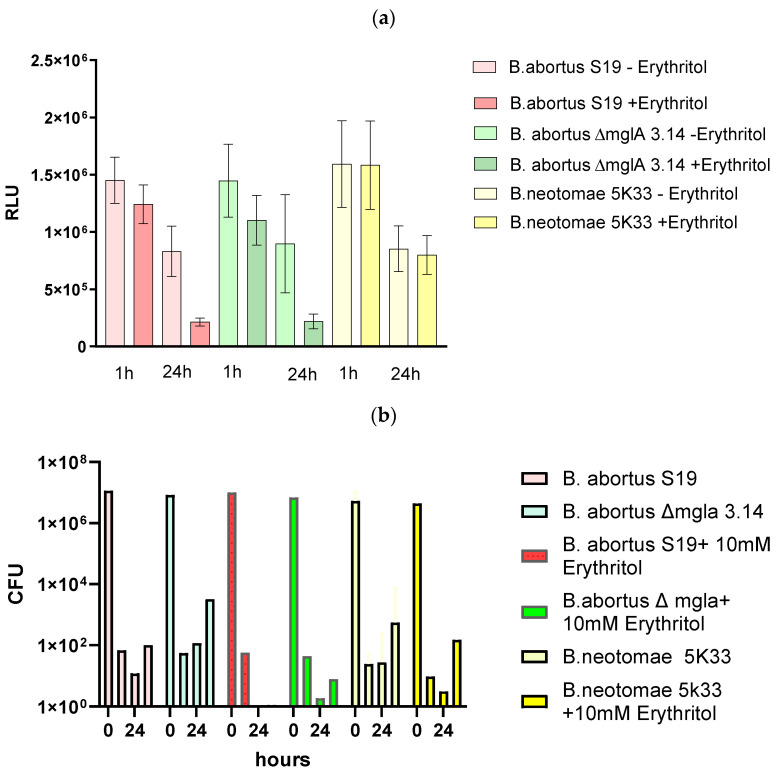
Erythritol effect on *B. abortus* S19A, *B. abortus* ∆mglA 3.14, and *B. neotomae* 5K33, after 1–24 h as determined via ATP content (**a**) and CFU on TSA (**b**).

*B. abortus* S19 is susceptible to erythritol, as previously known [3]. The nature of this visible inhibition is non-oxidative [3]. However, in this study, under the influence of erythritol, the formation of VBNC *Brucellae* was not observed (Figure 5, Table 5). This is because the luminescence values behaved similarly in *B.abortus* S19 and *B. abortus* S19 ΔmglA.

There were also no visible differences in the CFU counts obtained on TSA agar. Table 5 reports the luminescence and CFU values in the case of erythritol exposure in relation, i.e., quotas. The quotas did not increase, which indicates that relatively no more ATP was measured in comparison to the CFU counts in the cases of *B. abortus S19* and *B. abortus S19 ΔmglA.* Concluding from these findings, we assume that none of the (still alive, energy-possessing) bacteria were driven to the VBNC phase.

### 3.6. Effects of Isopropanol on CFU and ATP Content and ATP/CFU Ratios

**Figure 6:**
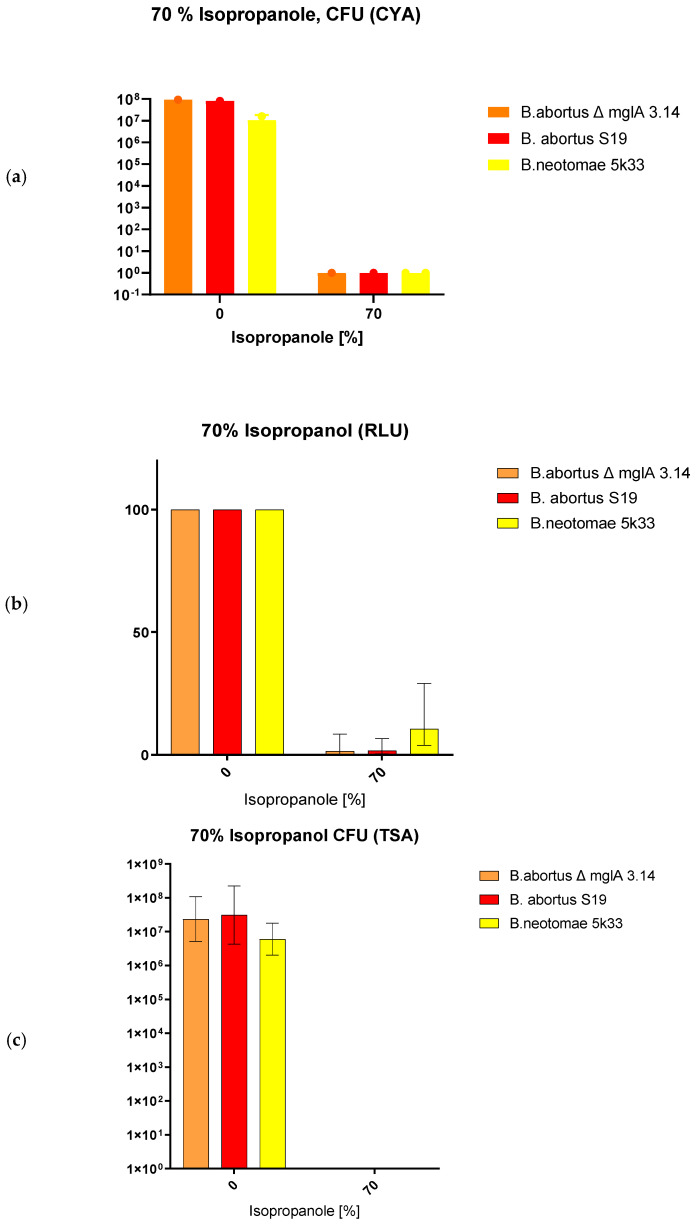
Effects of 70% isopropanol on *B. abortus* S19A, *B. abortus* ∆mglA 3.14, and *B. neotomae* 5K33 after 30 min of exposition as determined by ATP content (**b**), CFU on TSA (**c**), and CFU on CYE agar (**a**).

The non-oxidative effect of isopropanol on ATP content is indicated in Table 6 and Figure 6. The luminescence values behaved similarly for *B. abortus* S19 and *B. abortus* S19 ΔmglA. Table 6 shows a similar decline (but presence) of ATP in the strains tested. There was no visible difference in recovery by CYE in contrast to TSA agar. Additionally, Table 6 reports the luminescence and CFU values in case of isopropanol exposition in relation, i.e., quotas. The increase in quotas indicates that relatively more ATP was measured in comparison to the CFU counts in the cases of *B. abortus* S19 and *B. abortus* S19 ΔmglA. However, it is to be noted here that the effect of the isopropanol concentration used was so strong that no cultivability whatsoever could be detected in some variants of the assay, as also shown partly for bleach. Concluding from these findings, we assume that also under these non-oxidative isopropanol conditions, some of the bacteria were driven to the VBNC phase.

### 3.7. Determination of Membrane Integrity

**Figure 7:**
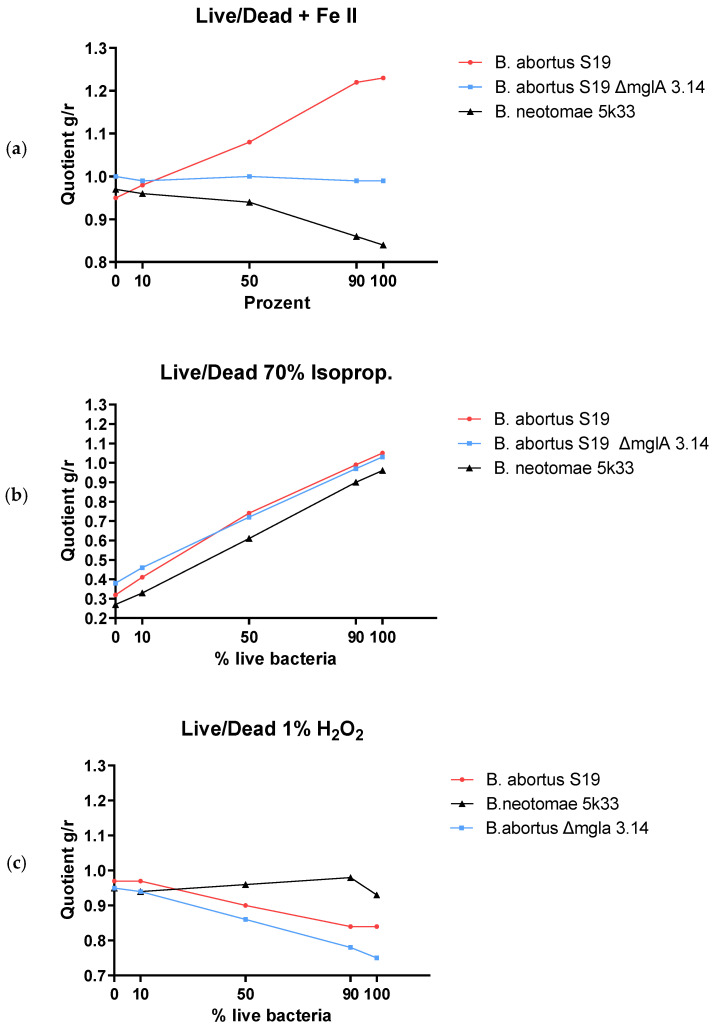
Membrane integrity as determined by live/dead experiments for *B. abortus* S19, *B. abortus* ΔmglA, and *B. neotomae* 5K33, (**a**) +Fe^2+^, (**b**) isopropanol, and (**c**) H_2_O_2_.

Figure 7 shows membrane integrity as determined by live/dead experiments for *B. abortus* S19, *B. abortus* ΔmglA, and *B. neotomae* 5K33 with (a) +Fe^2+^, (b) isopropanol, and (c) H_2_O_2_. The decline of g/r rations in the case of isopropanol (c), i.e., interruption of membrane integrity, indeed shows that this non-oxidative noxe causes real death in contrast to the already assumed VBNC in Section 3.6.

In Figure 7a,b, the relative stability of the g/r values under Fe^2+^ and H_2_O_2_, respectively, indicates that the bacteria shifted to the VBNC phase, as already mentioned in Section 3.1 and Section 3.2.

### 3.8. Bacterial Growth Comparison on TSA versus CYE Agar (Standard Plating Efficacy without Oxidative Stress)

In this study, the plating efficiency of CYE/TSA for *B. abortus* S19, *B. abortus* ∆mglA, and *B. neotomae* 5K33 was assessed. The charcoal-based CYE agar was superior for all *Brucella* strains tested, mostly *B. a.* S19 and the *B. a.* ∆mglA mutant. Because of that, the CYE agar was used in the stress experiments to minimize oxidative stress, i.e., to rescue *Brucella* back into the cultivable state.

## 4. Discussion

Among the investigated bacteria, *B. abortus* showed the highest susceptibility to oxidative stress induced by Fe^2+^, H_2_O_2_, or bleach, respectively, according to different assays (CFU (TSA/CYE), ATP (RLU-LUMI), and membrane integrity (live/dead)). Higher tolerance was found in *B. neotomae* 5K33.

In addition to our previous findings about a VBNC phase in *Brucella abortus S19* [1], our results presented here yield further evidence on the formation of a VBNC state in *Brucella*. The rise of the ATP (RLU-LUMI)/CFU quotients showed that under conditions of different types of oxidative stress (Fe^2+^, H_2_O_2_, bleach), the numbers of colony forming units dropped more quickly with the increase of ATP (RLU-LUMI) values.

We therefore conclude that the bacteria did not really die, i.e., they lost only cultivability but relatively preserved their ATP content. We proved that this hypothesis was right by plating on oxidative stress-reducing CYE agar. By means of that approach, we made cultivable a significant part of the former non-cultivable *Brucella*.

Our findings are in accordance with previous results for *Campylobacter* and *Helicobacter*, respectively, regarding a VBNC stage [8,18,19] and for (*Brucella-*related) *Agrobacterium tumefaciens* and *Rhizobium leguminosarum* [11] but also in general for VBNC bacteria [20].

This is the first report about the occurrence of VBNC (viable but nonculturable) *Brucella* confirmed on CYE agar.

We summarize that “viable but nonculturable” (VBNC) forms of *Brucella abortus S19* were detected on the basis of shifted ATP–CFU ratios and comparable growth on TSA versus CYE agar.

Various types of non-oxidative stress (selenite erythritol, selenite, isopropanol) did not show the above-described phenomena.

With respect to the findings in the study presented here, strategies for the epidemiology and control of the economically important brucellosis disease should also now be re-evaluated.

The immanent need for that is indicated by recent findings regarding the detection and distribution of VBNC/viable pathogenic bacteria in full-scale drinking water treatment plants and mechanisms underlying the effects of chlorination and UV disinfection on the VBNC state of *Escherichia coli* isolated from hospital waste waters [21,22]. With respect to the environmental perspectives, a complete list of actual challenges of VBNC bacteria was published by Bodor et al. [23].

## Figures and Tables

**Table 1:** Influence of “Low Iron” (0.2 mg/mL) conditions on the Brucella’s ATP content (RLU values) and cultivability (CFUs). Quota: RLU-LUMI/CFU (description of the VBNC phase).

Species/Strain	Mean RLU/LUMI Values (×10^6^)	RLU (%)	CFU/mL (TSA)	Quota RLU-LUMI/CFU (TSA)
*B. abortus* S19	1.1	100	5.5 × 10^6^	0.2
*B. abortus* S19 (+Fe^2+^)	0.9	73	5.5 × 10^4^	**1636**
*B. abortus* S19 ∆mglA	1.7	100	8.5 × 10^5^	0.2
*B. abortus* S19 ∆mglA (+Fe^2+^)	1.5	93	2.7 × 10^3^	**556**
*B. neotomae* 5K33	3.1	100	1.6 × 10^7^	0.2
*B. neotomae* 5K33 (+Fe^2+^)	3.4	110	4.9 × 10^6^	0.7

**Table 2:** Influence of H_2_O_2_ (0.5%) conditions on the Brucella’s ATP content (RLU values) and cultivability (CFUs). Quota: RLU-LUMI/CFU (description of the VBNC phase).

Species/Strain	Mean RLU/LUMI Values	RLU (%)	CFU/mL (TSA)	Quota RLU-LUMI/CFU(TSA)
*B. abortus* S19	2.0 × 10^6^	100	6.3 × 10^6^	0.3
*B. abortus* S19 +0.5% H_2_O_2_	4.5 × 10^6^	85.9	0	4.5 × 10^6^
*B. abortus* ∆mglA	1.8 × 10^6^	100	6.9 × 10^6^	0.3
*B. abortus ∆*mglA + 0.5% H_2_O_2_	1.9 × 10^6^	94	0.15 × 10^6^	12.7
*B. neotomae* 5K33	1.7 × 10^4^	100	23 × 10^6^	0.01
*B. neotomae* 5K33 + 0.5% H_2_O_2_	1.6 × 10^4^	93	4 × 10^6^	0.04

**Table 3:** Effects of 0.002% NaOCl conditions on the Brucella’s ATP content (RLU values) and cultivability (CFUs). Quota: RLU-LUMI/CFU (description of the VBNC phase).

Species/Strain	Mean RLU/LUMI Values	RLU (%)	CFU/mL (TSA)	Quota RLU/LUMI/CFU (TSA)	CFU/mL (CYE)	Quota RLU-LUMI/CFU (CYE)
*B. abortus* S19	1.1 × 10^6^	100	1.6 × 10^7^	0.07	5.0 × 10^7^	0.02
*B. abortus* S19 + 0.5% bleach	1.0 × 10^6^	91.5	0	**1 × 10^6^**	0	**1 × 10^6^**
*B. abortus* ∆mglA	3.2 × 10^6^	100	7.4 × 10^6^	0.4	7.3 × 10^7^	0.04
*B. abortus* ∆mglA + 0.5% bleach	2.9 × 10^6^	89.4	0	**2.9 × 10^6^**	0	**2.9 × 10^6^**
*B. neotomae* 5K33	2.8 × 10^6^	100	6.4 × 10^6^	0.4	1.5 × 10^7^	0.19
*B. neotomae* 5K33 + 0.5% bleach	2.8 × 10^6^	97.6	0	**2.8 × 10^6^**	1.9 × 10^3^	**1450**

**Table 4:** Effects of 10 mM selenite conditions on the Brucella’s ATP content (RLU values) and cultivability (CFUs on TSA and CYE). Quota: RLU-LUMI/CFU (description of the VBNC phase).

Species/Strain	Mean RLU/LUMI Values	RLU (%)	CFU/mL (TSA)	Quota RLU/LUMI/CFU (TSA)	CFU/mL (CYE)	Quota RLU-LUMI/CFU (CYE)
*B. abortus* S19 control	1.1 × 10^6^	100	1.3 × 10^7^	0.08	3.8 × 10^7^	0.03
*B. abortus* S19 + 10 mM selenite	9.0 × 10^5^	79.9	1.0 × 10^7^	0.09	4.1 × 10^7^	0.02
*B. abortus* S19 + 100 mM selenite	2.0 × 10^5^	18.0	6.2 × 10^6^	0.03	4.1 × 10^7^	0.005
*B. abortus* ∆mglA control	3.2 × 10^6^	100	6.7 × 10^6^	0.5	7.7 × 10^7^	0.04
*B. abortus* ∆mglA + 10mM selenite	2.1 × 10^6^	64.5	3.8 × 10^6^	0.6	3.6 × 10^7^	0.06
*B. abortus* ∆mglA +100 mM selenite	6.5 × 10^5^	20.3	1.6 × 10^6^	0.4	5.6 × 10^7^	0.01

**Table 5:** Effects of 10 mM erythritol conditions on the *Brucella*’s ATP content (RLU values) and cultivability (CFUs on TSA and CYE). Quota: RLU-LUMI/CFU (description of the VBNC phase).

**Species/Strain/Incubation Time** **1 h Erythritol**	**Mean RLU/LUMI (×10^6^)**	**RLU (%)**	**CFU/mL (TSA)**	**Quota RLU-LUMI/CFU (TSA)**
*B. abortus* S19	1.5	100	7.0 × 10^6^	0.2
*B. abortus* S19 (+ erythritol)	1.2	85.6	4.5 × 10^6^	0.3
*B. abortus* S19 ∆mglA	1.4	100	1.2 × 10^7^	0.1
*B. abortus* S19 ∆mglA (+ erythritol)	1.1	76.4	9.7 × 10^7^	0.01
*B. neotomae* 5K33	1.6	100	8.3 × 10^6^	0.2
*B. neotomae* 5K33 (+ erythritol)	1.6	99.2	1.1 × 10^7^	0.15
**Species/Strain/Incubation Time** **24 h Erythritol**	**Mean RLU/LUMI Values (×10^6^)**	**RLU (%)**	**CFU/mL (TSA)**	**Quota RLU/LUMI/CFU (TSA)**
*B. abortus* S19	0.83	100	9.8 × 10^8^	0.0001
*B. abortus* S19 (+ erythritol)	0.21	27.0	5.0 × 10^5^	0.4
*B. abortus* S19 ∆mglA	0.9	100	1.3 × 10^9^	0.001
*B. abortus* S19 ∆mglA (+ erythritol)	0.22	26.7	2.4 × 10^6^	0.09
*B. neotomae* 5K33	0.85	100	8.9 × 10^8^	0.001
*B. neotomae* 5K33 (+ erythritol)	0.80	94.2	6.3 × 10^8^	0.001

**Table 6:** Effects of 70% isopropanol conditions on the *Brucella*’s ATP content (RLU values) and cultivability (CFUs on TSA and CYE). Quota: RLU-LUMI/CFU (description of the VBNC phase).

Species/Strain	Mean RLU/LUMI Values	RLU (%)	CFU/mL (TSA)	Quota RLU-LUMI/CFU (TSA)
*B. abortus* S19	2.7 × 10^5^	100	3.1 × 10^7^	0.001
*B. abortus* S19 + isopropanol	4.5 × 10^3^	1.7	0	4.5 × 10^3^
*B. abortus* S19 ∆mglA	7.9 × 10^5^	100	2.4 × 10^7^	0.03
*B. abortus* S19 ∆mglA + isopropanol	1.3 × 10^4^	1.5	0	1.3 × 10^4^
*B. neotomae* 5K33	2.1 × 10^5^	100	6 × 10^6^	0.04
*B. neotomae* 5K33 + isopropanol	2.2 × 10^4^	4.5	0	2.2 × 10^4^

## Data Availability

Data supporting reported results are available by request from the author. For the moment these data are not deposited at publicly available datasets.
